# Environmental Enrichment Preceding Early Adulthood Methylphenidate Treatment Leads to Long Term Increase of Corticosterone and Testosterone in the Rat

**DOI:** 10.1371/journal.pone.0022059

**Published:** 2011-07-15

**Authors:** Avi Avital, Talya Dolev, Shlomit Aga-Mizrachi, Salman Zubedat

**Affiliations:** 1 Department of Psychology and the Center for Psychobiological Research, The Yezreel Valley College, Yezreel Valley, Israel; 2 Department of Neurobiology, The Weizmann Institute of Science, Rehovot, Israel; University of Queensland, Australia

## Abstract

Attention-deficit/hyperactivity disorder (ADD/ADHD) has been emerging as a world-wide psychiatric disorder. There appears to be an increasing rate of stimulant drug abuse, specifically methylphenidate (MPH) which is the most common treatment for ADHD, among individuals who do not meet the criteria for ADHD and particularly for cognitive enhancement among university students. However, the long term effects of exposure to MPH are unknown. Thus, in light of a developmental approach in humans, we aimed to test the effects of adolescence exposure to enriched environment (EE) followed by MPH administration during early adulthood, on reactions to stress in adulthood. Specifically, at approximate adolescence [post natal days (PND) 30–60] rats were reared in EE and were treated with MPH during early adulthood (PND 60–90). Adult (PND 90–92) rats were exposed to mild stress and starting at PND 110, the behavioral and endocrine effects of the combined drug and environmental conditions were assessed. Following adolescence EE, long term exposure to MPH led to decreased locomotor activity and increased sucrose preference. EE had a beneficial effect on PPI (attentive abilities), which was impaired by long term exposure to MPH. Finally, the interaction between EE and, exposure to MPH led to long-term elevated corticosterone and testosterone levels. In view of the marked increase in MPH consumption over the past decade, vigilance is crucial in order to prevent potential drug abuse and its long term detrimental consequences.

## Introduction

Methylphenidate (MPH; Ritalin) is commonly prescribed for the treatment of attention-deficit/hyperactivity disorder (ADD/ADHD), a psychiatric disorder that is more common during childhood and early adulthood. Treatment with MPH is generally effective in reducing symptoms associated with ADHD: inattentiveness, impulsivity, impaired working memory and hyperactivity [1]–[4]. Despite the widely recognized efficacy of MPH in the treatment of ADHD, little is known about the neural mechanisms that underlie the behavioral/cognitive actions of this drug. MPH inhibits dopamine and norepinephrine transporters, thereby increasing the extra-cellular concentration of these neurotransmitters [5]–[7].

Although ADHD can be difficult to diagnose [8], [9], the use of prescription stimulants for its treatment has increased over the past decade [10]–[12], particularly of MPH [13]–[15]. The latter findings may increase the likelihood that individuals who do not meet ADHD criteria, are being exposed to stimulants [10], [16] and could lead to extensive MPH abuse. The documented abuse consists of 6–9% non-medical consumption by individuals who do not have ADHD [17], [18], and 14% secondary abuse by ADHD patients who use their prescriptions for over consumption [19]. Importantly, MPH is also being widely consumed in an unsupervised manner for improving concentration and enhancing performance, or for recreational purposes among university students [20]–[24]. A report from 2006 indicates that more than 7 million people in the US have abused ADHD stimulants, and as many as 750,000 teenagers and young adults may show signs of addiction [25]. Yet, little is known about the long-term consequences of the exposure to MPH.

In trying to assess the time frame of adolescence in rat, it is difficult to characterize absolute boundaries [26]. Spear L.P. (2000) suggested that adolescence be considered between postnatal day (PND) 28–42 [26] while Spear L. (2000) suggested PND 28–55 [27]. Laviola (2003) suggested a wider definition between PND 21–60 [28], and recently Marco (2011) suggested PND 35–50 [29]. Thus, in order to encompass the various definitions, in the current study we have defined PND 30–60 as adolescence. Previous studies, utilizing a rat model, showed that the exposure to MPH during PND 20–35 decreased response to rewarding stimuli, increased depressive- and anxiety-like behaviors and enhanced corticosterone levels following restraint stress in adulthood [30]–[33]. However, in order to increase face validity of an animal model of MPH treatment in a non-ADD/ADHD condition, we aim to examine the long-term behavioral consequences of administration of MPH during early adulthood. Therefore, we postulate that a common human developmental path is comprised of the exposure to enriched environment (EE) during adolescence, a long term use/abuse of MPH during early adulthood, followed by coping with a stressful experience in adulthood. Importantly, we have focused on MPH treatment that occurred during early adulthood, since MPH has been widely unruly used by students for cognitive enhancement, weight loss or euphoric effects [20], [22], [24].

## Materials and Methods

### Animals

Male Wistar rats were purchased from Harlan (Jerusalem, Israel) and were reared at the institutional animal housing facility. Rats were housed four per cage (30L×30W×18H cm). Room temperature maintained at 23±1°C with 67% humidity with a 12∶12 day/night cycle (lights on at 06:00) and ad-libitum food and water access allowed. All behavioral tests and manipulations were held between 07:00 and 17:00. This study was carried out in strict accordance with the recommendations of the Guide for the Care and Use of Laboratory Animals of the National Institutes of Health. The protocol was approved by the Committee on the Ethics of Animal Experiments of the Israel Ministry of Health (Permit Number: IL-09-06-040). All efforts were made to minimize animal suffering.

### Procedure

**Figure 1:**
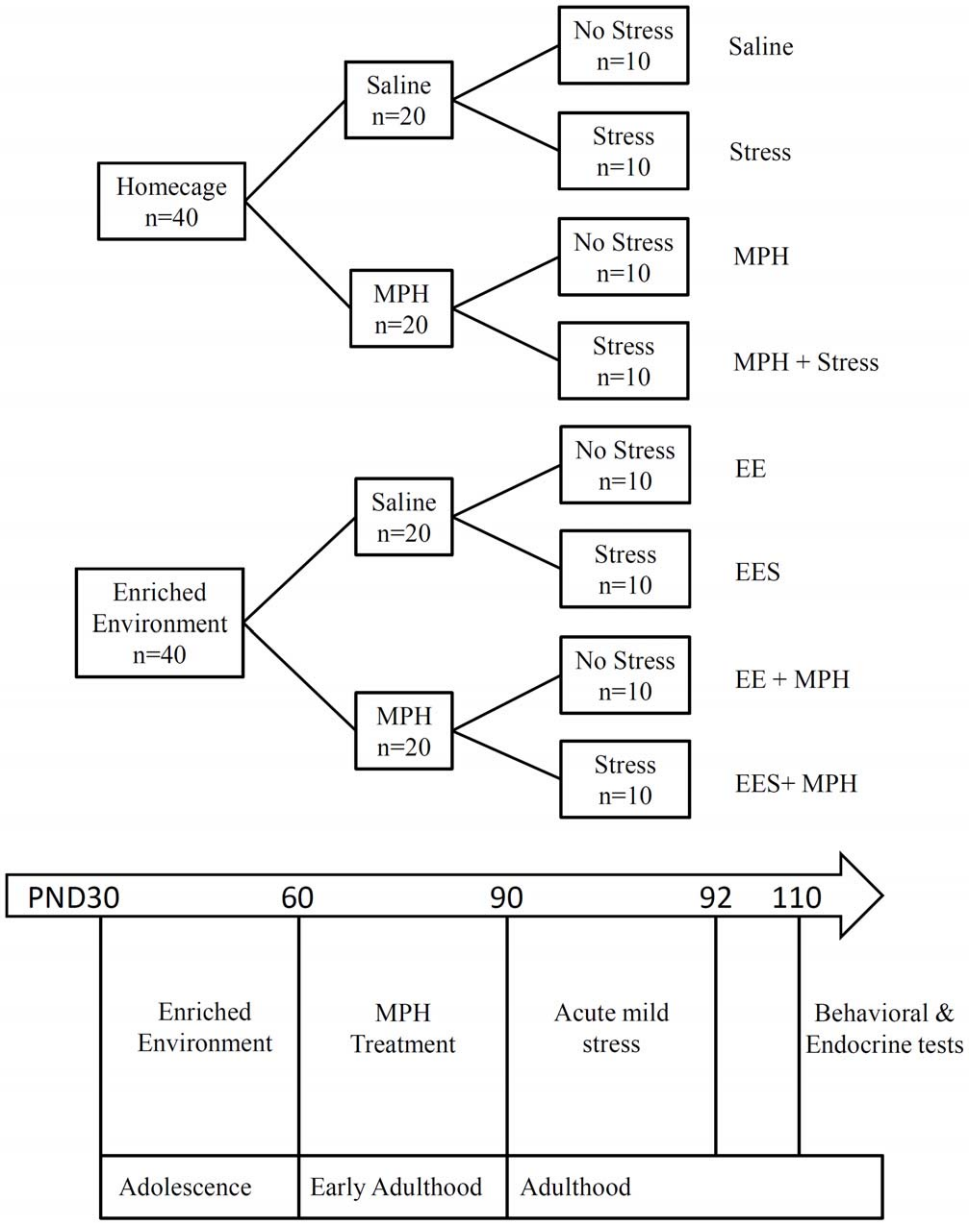
Schematic diagram of experimental design and procedures.

Between PND 30–60 (adolescence) rats were kept under enriched environment (EE) conditions. During early adulthood (between PND 60–90) rats were treated with MPH. Finally, in order to examine ability to cope with mild stress in adulthood, rats were exposed to stress (acute swim, elevated platform and restraint) during three consecutive days (PND 90–92) (Figure 1). To evaluate the long-term behavioral consequences, starting at PND 110, all rats went through behavioral tests (i.e; open field, sucrose preference test and pre-pulse inhibition). Rats were decapitated twenty-four hours after the behavioral tests and trunk blood samples were collected for hormonal analysis (Figure 1).

### Enriched Environment

Between PND30 and 60, forty rats were kept under enriched environment conditions, 8 rats interacted socially per customized cage (80L×50W×50H cm) with various objects (e.g. running wheel, stairs, tubes, Lego cubes, wood parts, hanging items, etc.). Each week the objects were replaced with new ones for subtle novelty. The cage also contained an extra elevated (25 cm) surface (20L×50W cm) that was accessible via stairs. In addition, a sandbox (20L×10W cm) was included in the main floor; in order to provide textural diversity within the cage.

### Drug treatment

Methylphenidate hydrochloride (Sigma-Aldrich, St. Louis, USA) was dissolved in a sterile saline solution. Rats were injected with methylphenidate hydrochloride (randomly 4 days per week/2.7 mg/Kg body weight/intraperitoneally) or with saline, between PND 60–90.

### Stress

Rats were exposed to the following stressors during 3 consecutive days (PND 90–92).

#### Acute Swim

At PND 90, rats were allowed 15 minutes to swim in a squared water (23±1°C) tank: 38×30 cm, water depth: 60 cm [34], [35].

#### Elevated Platform

At PND 91, rats were placed on a 50 cm high, 10 cm diameter platform three times for 30 minutes each time with 1 hour spent in a resting cage between periods on the platform [36].

#### Restraint

At PND 92, rats were placed in a radial-shaped restrainer (6 cm height) according to the exposure regime of the elevated platform (modified from [37]).

### Behavioral Tests

All tests were carried out in a dimly lit room (50 lux) with stable temperature 24±1°C.

#### Open Field Test

The open field is made of a black lusterless Perspex box (100L×100W×40H cm). Rats were placed in the corner of the open field (facing the wall). Their behavior (i.e; locomotor activity and freezing) was videotaped for 5 min by a CC TV Panasonic camera with post-recording analysis performed using Ethovision XT software (Noldus, Wageningen, The Netherlands).

#### Pre-Pulse Inhibition (PPI)

The PPI test is held in a ventilated sound proof box (Campden instruments, UK) and aims to examine the function of the sensorimotor gating. The session (a total of 80 trials) started with 3 min acclimatization period with a 57 dB background noise level that was delivered continuously throughout the test session. To evaluate the startle response, the first and last ten trials consisted of single 40 ms, 120 dB “pulse-alone” startle stimuli. These trials were used to obtain a measure of habituation in response to repeated delivery of the startling stimuli (inter-trial-interval 1 min). The middle 60 trials were comprised of random delivery of ten “no stimuli” trials, during which no stimuli are delivered, ten “pre stimuli” (at 59, 61, 65, 69, 73, 78 or 85 dB), and forty “pre pulse” trials. The latter trials, consisted of a single 120 dB pulse preceded (80 ms interval) by a 20 ms “pre pulse” of 2, 4, 8, 12, 16, 21 or 28 dB above background (i.e., 59, 61, 65, 69, 73, 78 or 85 dB). Finally, PPI was calculated as a percent score that reflects the ratio between the inhibited (“pre pulse” trial) response, and the individual startle (“pulse-alone”) response: [100−(max response to “pre pulse” trial/max response to “pulse alone” trial×100)].

#### Sucrose Preference Test

During the acclimatization period (1 week), rats were allowed to consume 1% (w/v) sucrose or tap water, in order to overcome neophobia. Moreover, they were under water limitation (access allowed for 4 h a day). On the 6^th^ day the amount of total liquid consumption was assessed (over a period of 4 h), to detect general differences in liquid consumption. Following acclimatization period, on the 8^th^ day, the test was carried out individually for each rat: following a 16 h period of water limitation, two drinking bottles, identical to the home cage water bottles, were inserted into the cage through the metal mesh top cover. The bottles, one containing a 10% sucrose solution and the other water, were weighed just before the test and immediately following its completion (after 4 h) and the sucrose preference which indicates natural reward was calculated. The relative positioning of the bottles providing sucrose and water was reversed after two hours, in order to prevent the development of side preference.

### Corticosterone and Testosterone

Twenty-four hours after the behavioral tests, rats were decapitated. To avoid circadian variability, all decapitations were performed between 11–12 a.m., when plasma hormones concentration is relatively low. Blood samples were centrifuged (2000×g at 4°C for 20 min), serum was collected and stored at −80°C until assayed. Serum corticosterone and testosterone levels were assessed using commercial ELISA kits (AssayPro, St. Charles, MO, USA) according to the manufacturer's instructions.

### Statistical Analysis

Data were analyzed for statistical significance using two-way ANOVA with group and treatment as main factors (4×2). For analyzing body-weight and PPI we used ANOVA for mixed design, with group as between subject's factor and test timing or pre-pulse intensity as within subject's factor. In order to further explore the main effects, a Post-Hoc Tukey test was performed. A result was significant when p<0.05. All tests were calculated as two-tailed with SPSS V17.0. Results are presented as means ± standard error of the means (SEM).

## Results

### The long-lasting effects of MPH on locomotion and freezing behaviors

**Figure 2:**
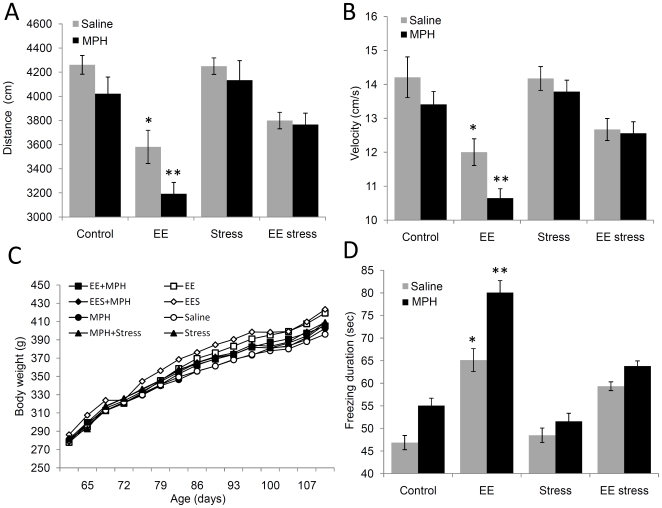
The effect of MPH on locomotor activity. A significant difference in distance (A) and velocity (B) were detected between the groups in the open field test. Rats exposed to MPH following EE showed the lowest distance and velocity. These effects were not related to differences in body weight (C). MPH treatment significantly increased freezing duration (D) compared to saline. Following EE, MPH led to the highest freezing duration. * *P*<0.0001 versus control; ** a: *P*<0.022, b: *P*<0.008, c: *P*<0.0001 versus EE saline; n = 9 to 10 per group.

A significant difference in locomotor activity measured by distance (Figure 2A) and velocity (Figure 2B) was detected between the groups [F(3, 71) = 25.4, *P*<0.0001)]. We found that adolescence EE led to a long term decrease in distance and velocity (*P*<0.0001). Surprisingly, MPH administration following adolescence EE intensifies these effects over time (*P*<0.022; *P*<0.008, respectively). Interestingly, the exposure to a brief stress (EE+Stress) in adulthood moderates these alterations. In order to exclude the effect of body weight on locomotor behavior, indeed an insignificant difference in body weight gain (Figure 2C) between all groups was found [F(7, 71)<1].

Measuring freezing behavior (Figure 2D), a significant difference was detected between the groups [F(3, 71) = 75.83, *P*<0.0001]. The highest freezing level was detected in the EE group (*P*<0.0001), while the EE followed by stress (EES) demonstrated a significant increase compared to both control and stress groups (*P*<0.0001). In addition, MPH was associated with significantly higher freezing in both control (*P*<0.001), EE (*P*<0.0001) and EES (*P*<0.006) groups.

### MPH modulates the response to rewarding stimulus (hedonia)

**Figure 3:**
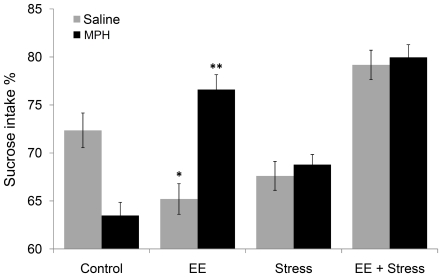
The effect of MPH on sucrose preference test. Considerable variation in sucrose intake was observed between the groups. While significant long term anhedonia was detected in the EE group, MPH treatment following EE significantly recovers this effect. * *P*<0.019 versus control; ** *P*<0.0001 versus EE saline; n = 9 to 10 per group.

In order to test the response to rewarding stimulus, we have tested hedonia/anhedonia rate by measuring sucrose intake (Figure 3). A significant diversity in sucrose preference was observed between the groups [F(3, 71) = 31.57, *P*<0.0001]. Complementarily to the highest freezing level observed in the EE group, a significant long term anhedonia was detected (*P*<0.019). Fascinatingly, MPH administration has led to anhedonia in the controls (*P*<0.001), while in the EE group MPH significantly shifted to increased hedonia (*P*<0.0001). Finally, the EES group showed a significant hedonia (*P*<0.0001) as compared with either the EE or stress groups, that was not affected by MPH administration.

### MPH detrimental effects on Pre-Pulse Inhibition following adolescence EE or preceding stress in adulthood

**Figure 4:**
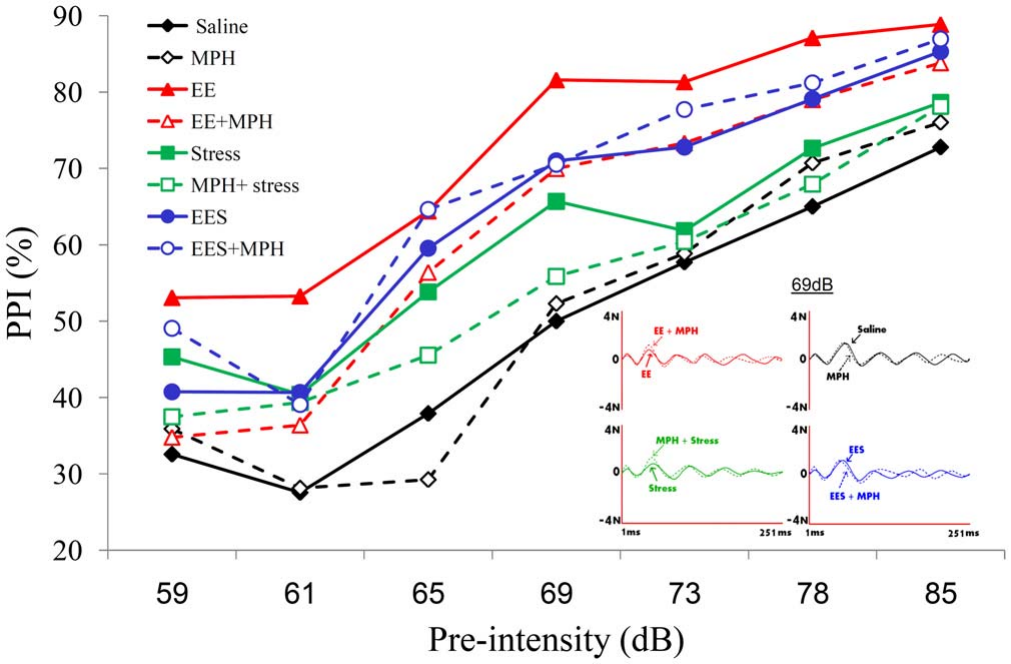
The effect of MPH on PPI. Significant differences in PPI scores were observed. EE led to the highest PPI compared with all groups (*P*<0.0001). Following MPH treatment, the EES group showed higher PPI compared with control (*P*<0.0001) and stress (*P*<0.001) groups. A panel of representative traces demonstrate the differences in maximal response inhibition (at pre-intensity of 69 dB) of all four groups, with and without MPH. n = 9 to 10 per group.

Pre-Pulse Inhibition test (PPI) is a neurological phenomenon (measured also in human subjects) in which a weaker acoustic pre-pulse, inhibits the reaction to a subsequent strong startling pulse. The reduction of the amplitude of response reflects the ability of the nervous system to temporarily adapt to a strong sensory stimulus when a preceding weaker signal is given. We found (Figure 4) a significant distinction in PPI along different pre- intensities (59 db to 85 db) across all groups [F(6, 30) = 459.42, *P*<0.0001) and between the groups [F(3, 35) = 161.12, *P*<0.0001]. The exposure to adolescence EE has led to a long term beneficial effect on PPI (*P*<0.0001), while stress following EE has lessened this increase (*P*<0.019). When administrated following EE (EE+MPH) or prior to stress (MPH+stress), MPH had a long term deteriorating effect on PPI (*P*<0.022; *P*<0.004, respectively). However, by itself (MPH group) or in the EES group, MPH seemed to have only minor effect.

### MPH leads to a long-term increase of Corticosterone and Testosterone depending on the developmental context

**Figure 5:**
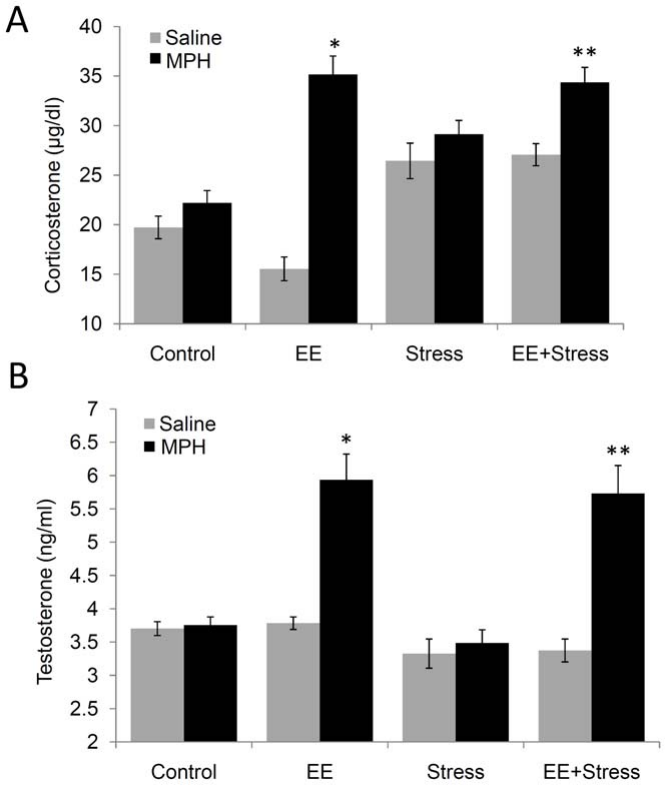
The effect of MPH on CORT and TST levels. A significant difference in CORT (A) and TST (B) levels was observed. MPH treatment significantly increased CORT level in the EE and EES groups (* P<0.0001; ** P<0.002 compared with their respective controls). TST level was similar across all groups, while MPH treatment increased TST level in both EE and EES groups (* P<0.001; ** P<0.012 compared with their counterpart controls). n = 9 to 10 per group.

In trying to depict the endocrine mechanism mediating the effects of MPH in the context of the developmental approach, previous studies found that EE lowered baseline corticosterone (CORT) level [38] or did not influence its level at all [39], while MPH (similar to hyper-arousal following stress) was found to increase CORT level [30], [33], [40], [41]. To clarify the aforementioned behavioral effects, we measured serum CORT level (Figure 5A) and found a significant difference between the groups [F(3, 56) = 51.019, *P*<0.0001]. Without MPH, a long term significant CORT reduction was found in the EE group (*P*<0.026) while in the stress and the EES groups CORT was elevated (*P*<0.004). Interestingly, MPH administration increased CORT level (more than 152%) in the EE (*P*<0.0001) and EES (*P*<0.002) groups.

Finally, MPH is known to affect aggression [42], [43]. Specifically, it has been suggested that children with ADHD generate more aggressive responses to provocation and that this may be exacerbated by administration of MPH [42]. This led us to examine the endocrine correlate of aggression, by measuring Testosterone (TST) serum level [44]–[47]. A significant variation in TST level (Figure 5B) was observed between the groups [F(3, 56) = 14.12, *P*<0.0001]. Surprisingly, we found a robust long-term increase (more than 154%) in the EE and EES groups treated with MPH (*P*<0.001 and *P*<0.012 respectively).

## Discussion

Psychostimulants have proven to be an effective pharmacotherapy for ADD/ADHD, with MPH being the preferred medication for this neuropsychiatric disorder [48]. Moreover, in several pediatric populations more than half of those treated with MPH do not meet the criteria for attention disorders [10], [16], [49].Given the prevalence of prescribed use of MPH, it is surprising that only few studies have utilized a developmental approach to explore the long-term effects of MPH. These studies showed enduring behavioral alterations in adult animals as a consequence of repeated therapeutic dose of MPH exposure during their juvenile period [30]–[33]. However, in order to increase face-validity, one should take into consideration the importance of timing and the diversity of life experiences. The latter has major importance in light of the raise of MPH abuse among college students [20], [22], [24]. Thus, we aim to examine the long-lasting behavioral and endocrine consequences of the exposure to environmental enrichment (during adolescence), long term MPH treatment (during early adulthood) and stress (during adulthood).

Our findings show no significant effect of MPH administration on body weight, similar to ADHD children treated with MPH [50]. However, we found that adolescence EE lead to a long term decrease in locomotor activity, while MPH increased these effects. In line with these findings, we also found that EE increased freezing duration compared with control group. It should be noted that MPH increased freezing in both control and EE groups. To this end, Bolanos et al (2003) found that pre-pubertal MPH treatment also decreased locomotor activity [30]. Moreover, Britton et al (2007) found enhanced anxiety-like behaviors in adult rats caused by early developmental MPH treatment [51].

However, in our study we suggested to take into account also the environmental context in which the individual grows, since we postulate that many children that were raised under EE consume MPH mainly during early adulthood. In support of this idea, while some reports indicate that EE has an anxiolytic effect [39], [52], [53], others report that the exposure to EE pre-pubertally may serve as anxiogenic factor [54], [55]. A wide range of data in the literature suggests that EE has beneficial effects on various behavioral parameters in rodents. However, the magnitude of these effects and their persistence after the cessation of enrichment vary markedly across studies according to the developmental stage in which the exposure occurred (starting pre-weaning [56] up to old age [57]) and to the duration of enrichment (from a couple of weeks [58], [59] to up to a year [60]) [61].

In addition, EE has been suggested to recover some of the deteriorating effects of early exposure to stress [62]–[64]; however the preceding effects of EE on the reaction to stress later in life are less investigated. In support of our findings, Amaral et al (2008) also found that 4 weeks of EE post weaning decreased locomotion activity in the open field test [61].

Since it was documented that MPH treatment during pre-adolescence often produces side effects such as decreased response to rewarding stimuli (i.e; increased depressive-like behaviors) [13], [65], [66], we have measured anhedonia utilizing the sucrose preference test. Similar to the aforementioned findings, we also found that MPH led to anhedonia in control animals. However, the exposure to MPH following adolescence EE, has recovered the anhedonia observed to the controls level. Interestingly, the combination of adolescence EE and stress in adulthood, both in MPH-treated and -untreated animals, led to increased hedonia. Complementarily, EE+MPH (with or without stress) led to the highest hedonia level, i.e; the preference of the rewarding stimulus.

Since treatment with MPH is generally effective in reducing such symptoms associated with ADHD as inattentiveness [2] and it also has been found that EE improved attentive abilities [67], [68], we examined in our non-ADHD rats the attention ability as reflected by the PPI test. Prepulse inhibition (PPI) of the acoustic startle reflex refers to the reduction in the magnitude of the startle reflex when a loud startling stimulus (termed the “pulse”) is preceded by a quieter nonstartling stimulus (termed a “prepulse”) at short stimulus onset asynchronies [69], and represents an operational measure of sensorimotor gating [70], [71]. However, other studies have been shown that PPI is not a completely automatic process; it occurs involuntarily but can be modulated by controlled attentional processes and has been used as a tool for investigating attention [72], [73].

We found that compared with the control group, the exposure to MPH didn't significantly alter PPI. Thus, it appears that allegedly MPH has no effect in “normal” conditions. However, while the exposure to EE led to the highest PPI rate, subsequent MPH treatment significantly impaired attentiveness. Similarly, the exposure to mild stress was associated with intermediate PPI levels (higher than the control but lower than EE), that were reduced by the presence of MPH.

Taken together, the results of the PPI test indicate that when MPH treatment is superfluous (i.e; without clear indication of ADD/ADHD), it may bare no effect or even paradoxly impairs attentiveness. It should be noted that previous studies which evaluated behavioral alterations following the exposure to pre-pubertal MPH, did not measure changes in attentiveness [30]–[33].

In order to better understand the behavioral consequences of MPH treatment, we first measured serum CORT level. It has been shown previously that MPH treatment leads to increased levels of CORT [30], [33]. Likewise, we found that long term MPH administration elevated CORT to a marked degree, but only in the EE and EES groups. Thus, we suggest that adolescence EE together with early adulthood MPH abuse may act as a predisposition for the development of possible stressful behaviors in adulthood.

Studies of the hypothalamic-pituitary-adrenal (HPA) axis and the hypothalamic-pituitary-testicular (HPT) axis revealed a reciprocal relationship between these two endocrine pathways, in which CORT increases the negative feedback effects of TST [74]–[77]. Therefore, the observed high level of CORT subsequent to MPH treatment is expected to be accompanied by a low level of TST. Consequently, we found a surprising elevation of TST level following MPH administration in the EE and EES groups. Hence, adolescence EE followed by MPH treatment during early adulthood, impaired the expected natural level balance between the two hormones, and overall led to long term hormonal agitation.

Furthermore, TST was found to elicit aggressive behavior in children, juvenile and adult males, in both rats and humans [78]–[81]. However, among ADHD treated patients, MPH was found to reduce aggression [43], [82]. Together with our finding, a “pro-aggression” effect of MPH may be observed when given to subjects without ADD/ADHD (i.e., abuse).

Thus, we suggest that a distinction should be made between justified medical consumption of MPH, which leads to beneficial effects, and MPH abuse that may lead to increased arousal and aggression. Additionally, it raises ethical questions regarding reports on the use of MPH as a “Cognitive Enhancing drug” in non-medical situations (i,e; shift work, military personnel, etc.) [21].

Our study demonstrates for the first time the long term effects of MPH treatment (in a non-ADD/ADHD condition) in the context of various life experiences. Following adolescence EE, MPH had deteriorating behavioral and hormonal effects. Adding stress in adulthood restored only the behavioral effects, while both corticosterone and testosterone levels remained high. Therefore, in non-ADHD subjects, MPH allegedly has no overt (behavioral) effects, while covertly (hormonal) it has robust and long lasting effects. Future studies may further the hormonal effects by exploring changes in behaviors related to aggression and response to rewarding stimuli (such as cocaine self administration).

Thus, cautious decision making while prescribing MPH is required in order to prevent its harmful social long-term consequences.
